# Depression, anxiety, and academic performance in COVID-19: a cross-sectional study

**DOI:** 10.1186/s12888-022-04062-3

**Published:** 2022-06-30

**Authors:** Francisco José Barbosa-Camacho, Olaya Moramay Romero-Limón, Juan Carlos Ibarrola-Peña, Yolanda Lorelei Almanza-Mena, Kevin Josué Pintor-Belmontes, Verónica Alexandra Sánchez-López, Jonathan Matías Chejfec-Ciociano, Bertha Georgina Guzmán-Ramírez, José Héctor Sapién-Fernández, Mario Jesús Guzmán-Ruvalcaba, Rodrigo Nájar-Hinojosa, Itzel Ochoa-Rodriguez, Tania Abigail Cueto-Valadez, Andrea Estefanía Cueto-Valadez, Clotilde Fuentes-Orozco, Ana Olivia Cortés-Flores, Roberto Carlos Miranda-Ackerman, Guillermo Alonso Cervantes-Cardona, Gabino Cervantes-Guevara, Alejandro González-Ojeda

**Affiliations:** 1 Unidad de Investigación Biomédica 02. Hospital de Especialidades, Centro Médico Nacional de Occidente, Instituto Mexicano del Seguro Social, Guadalajara, Jalisco México; 2 Hospital Civil de Guadalajara “Fray Antonio Alcalde”, Universidad de Guadalajara, Guadalajara, Jalisco Mexico; 3 Hospital General de Zona y Medicina Familiar number 2, Instituto Mexicano del Seguro Social, Nuevo León Monterrey, México; 4 Hospital General “Dr. Manuel Gea González”, Ciudad de México, Mexico; 5 Instituto Nacional de Ciencias Médicas y Nutrición “Salvador Zubirán”, Ciudad de México, Mexico; 6 Hospital San Javier, Unidad de Cuidados Intensivos, Guadalajara, Jalisco México; 7 Departamento de Disciplinas Filosófico, Metodológicas e Instrumentales, Centro Universitario de Ciencias de La Salud, Universidad de Guadalajara, Guadalajara, Jalisco Mexico; 8 Departamento de Bienestar y Desarrollo Sustentable, Centro Universitario del Norte, Universidad de Guadalajara, Colotlán, Jalisco México

**Keywords:** Anxiety, COVID-19, Depression, Educational needs assessment, Educational measurements, Mental health

## Abstract

Depression and anxiety are common after months of social isolation, and they can have a negative impact on anyone's quality of life if they are not treated promptly and appropriately. The aim of this study was to determine if the change to online modality courses and the presence of depression or anxiety symptoms during the COVID-19 pandemic was associated with a difference in the college student’s academic achievement. This study was a cross-sectional survey in which we used the Patient Health Questionnaire-9 (PHQ-9) and the General Anxiety Disorder-7 (GAD-7). Also, we examined the students' perceptions of their academic performance using the Academic Self-Concept Scale (ASCS). A total of 610 students responded to the survey. The average score on the Academic Self-Concept Scale was 2.76 ± 0.35, the students presented a risk of 61.5% for possible depressive disorder and 52.1% for possible generalized anxiety disorder. The intensity of depression and anxiety symptoms had a significant effect on Academic Self-Concept Scale scores (*p* < 0.001 and *p* < 0.05, respectively). The findings indicate that the COVID-19 pandemic has had a direct effect on students' mental health and academic performance.

## Educational impact and implications statement

The present study suggests that the change in educational modality in college students during the COVID-19 pandemic has various psychological consequences such as anxiety, depression, and stress, due to strict social isolation.

## Background

"I never teach my pupils; I only attempt to provide the conditions in which they can learn." – Albert Einstein.

After months of strict social isolation, it's unsurprising that the prevalence of depression and anxiety increased worldwide, negatively impacting the quality of life of everyone affected [1, 2]. Fear and stress can affect the prefrontal cortex, amygdala, and hippocampus's functionality, these disturbances can be expressed as erratic executive functions and appear as memory loss, low retention, or rapid changes in attention and decision-oriented objectives [3]. A similar situation occurred after the SARS pandemic in 2003 when survivors were diagnosed with posttraumatic stress syndrome, depression, anxiety disorders, and, to a lesser extent, obsessive–compulsive disorder, even four years after the outbreak [4]. The COVID-19 pandemic's various levels of stress and fear can influence the human psyche. A study on the Mexican population reported that during the initial phase of the COVID-19 epidemic, the reported number of anxiety and depression episodes increased by up to 51% and 86%, respectively [5]. Furthermore, major outbreaks of infectious diseases, such as COVID-19, have been linked to psychological distress [1], fear [6–8], anxiety [9], and even a novel disorder called coronaphobia [10, 11].

The COVID-19 pandemic is a unique scenario that has compelled governments to build plans to ensure social security and economic stability, as well as improvising techniques for responding to, adapting to, and overcoming daily barriers that allow the population to live as normally as possible [12, 13]. During the pandemic, the education process has been altered by different factors such as stress, depression, anxiety, and social isolation. Additionally, fear of contagion, the constant barrage of "bad news," changes in sleep patterns or eating habits, an increase in sedentary behavior, and poor social interaction all have a negative impact on our daily lives [14–17].

One fundamental challenge in this pandemic is the need to continue providing education in public and private academic institutes. Since most schools throughout the world were closed from March to the fall of 2021 [18–20], schools and universities continued to give classes online through platforms like Skype, Google, and Zoom [21]. Online education has grown in popularity over the last two decades because of its advantages over face-to-face education. Some studies report that students enjoy the online modality and have even presented better performance than their face-to-face counterpart [22–24]. A pre pandemic study compared the grades of a sample of medical students who took a preparation course that alternated between live and prerecorded classes [25]. The authors discovered similar results for both groups, implying that distance learning could partially replace traditional classroom instruction, although no students received exclusively live or video lectures.

However, in the context of the current crisis, the rapid incorporation of virtual education has distracted focus away from the modality's primary issues [26]. While transitioning from classroom-based instruction to online delivery is not difficult for students in most circumstances, being quarantined can be stressful and frustrating, limiting effective learning. Given the complexity of the present pandemic scenario, we were interested in how the shift to online education affects students' perspectives of their learning and performance.

## Methods

### Aims

This study aimed to explore whether the presence of mental health disorders, such as depression or anxiety related to the COVID-19 pandemic, was associated with a difference in their perception of their academic performance. As a secondary aim, this study aimed to identify the college students' preference for class modality (classroom or online).

### Study design

This study was a cross-sectional survey study in which we used the Academic Self-Concept Scale (ASCS) [27], the Patient Health Questionnaire-9 (PHQ-9) depression screening scale, and the General Anxiety Disorder-7 (GAD-7) screening scale. We asked the participants about their gender, age, their preference for teaching modality (classroom or online), and whether they perceived any differences in their performance or grades during the pandemic. The perceived differences in their performance or grades were assessed using a self-report 1-item question in which participants were asked to rate the extent to which their performance/grades were different from before and after the pandemic. The study’s independent variables were the PHQ-9 scale score, GAD-7 scale score, and ASCS scores. On the other hand, the study’s dependent variables were age, gender, self-perceived performance and grade differences.

### Participants

The survey was sent to students from public and private universities in Guadalajara, Jalisco, from April to June 2020 through the social media accounts or acquaintances of the authors.

Using Kelsey formula: α: error I (0.05), β: error II (0.80), p_0_: prevalence of anxiety in general population (0.11), p_1_: prevalence of anxiety in Jalisco population (0.16), r: relation (1:1), *p* = $\frac{p0+rp1}{r+1}$ (0.33), *n* = sample size.

$$\frac{{\left(Z\alpha +Z\beta \right)}^{2}*p\left(1-p\right)\left(r+1\right)}{{r\left(p0-p1\right)}^{2}}$$

The sample size was based on anxiety prevalence, in accordance with Baxter et al. [28] the general population present an anxiety prevalence of 11% around the world, however in a study by Ojeda-Torres et al. [29], the prevalence of anxiety symptoms in Jalisco is 16%. A minimum sample size of 366 individuals was calculated using an α error of 0.05 and β of 0.20. When adding a 20% to cover up incomplete surveys or excluded cases, 440 participants was calculated. Participants were invited to send the survey to other college students to obtain a snowball sampling effect. A total of 900 students were invited to the study, from which 720 agreed to answer the survey (non-response rate of 20%). However, 110 surveys were eliminated due to being incomplete resulting in 610 students. The inclusion criteria were > 18 years old and enrollment in any college online course. The exclusion criteria were not attending an online course, not being registered in a university undergraduate or graduate course, and all participants with a previous diagnosis of anxiety, depression, or any mood disorder. To ensure that the participants were enrolled in a university course, the survey included verifiable identifying information, such as the program they were studying, university, and enrollment number. This information would then be corroborated with the corresponding authorities. Any survey with incorrect or missing information was excluded from the study.

### Instruments

#### Academic Self-Concept

To evaluate academic performance, we used the ASCS [27, 30, 31]. This instrument is a 20-item inventory where the participant is presented with statements such as “I can follow the lectures easily”, “If I work hard, I think I can get better grades” or “I often feel like quitting the degree course”. The participant must answer each statement with a Likert scale of four options from "strongly disagree" (0 points) to "strongly agree" (3 points) which are used to explore a student's perception of their academic performance. The scoring is divided into two subscales: Confidence, which evaluates the student's academic competence through the course, and Effort, which evaluates the student's commitment and interest in the course. We found an acceptable internal validity (Cronbach α: 0.702).

#### Depressive symptoms

To evaluate depression, we used the PHQ-9 [32], a nine-question inventory used to identify the presence and intensity of depression symptoms. The participants are presented with nine statements, each with a Likert scale of 4 items ranging from “Not at all” (0 points) to “Nearly every day” (3 points) about how often they have been presenting symptoms over the past two weeks. No risk of depression or minimal risk was defined when a score of 0 to 4 was obtained, mild depression symptoms when it was 5 to 9, moderate depression symptoms with a score of 10 to 14, moderately severe depression symptoms from 15 to 19, and severe depressive symptoms with a score greater than 20. Patients with a score ≥ 10 are more likely to be diagnosed with depression by a mental health professional [33]. We found a great internal validity (Cronbach α: 0.890).

#### Anxiety symptoms

We used the GAD-7 questionnaire to assess anxiety [34]. This seven-item questionnaire assesses the presence and intensity of anxiety disorders. The participants are presented with seven statements, each with a Likert scale of 4 items ranging from “Not at all” (0 points) to “Nearly every day” (3 points) about how often they have been presenting symptoms over the past two weeks A score of 5–9 represents mild anxiety, 10–14 moderate anxiety, and scores greater than 15 represent severe anxiety. Similar to PHQ-9, patients with a score ≥ 10 are more likely to be diagnosed with generalized anxiety disorder [35]. We found a great internal validity (Cronbach α: 0.888).

### Data analysis

The data analysis was performed using SPSS Statistics software. The descriptive analyses included proportions, means, and standard deviations. The inferential analysis was performed using the chi-squared test, analysis of variance (ANOVA), and Student's *t-test*. Additionally, post hoc analysis was performed using Tukey honestly significant difference (HSD) and Bonferroni test. A probability level of *p* < 0.05 was considered to be significant. All variables were assessed with Levene’s Test for Equality of Variances, assuring that all variables had a parametric distribution.

### Ethics approval and consent to participate

Written consent was obtained from each participant. The surveys were anonymous to guarantee the confidentiality of each participant. This study complies with national committees' ethical standards on human experimentation and the Helsinki Declaration of 1975, as revised in Fortaleza, Brazil 2013. The study protocol was submitted to ClinicalTrials.gov and registered with the identifier NCT04420416 on 09/06/2020.

The National Ethics Committee and the National Scientific Research Committee authorized the study protocol with the identifier: F-2021–1301-227 on 05/07/2020.

## Results

A total of 610 students were included in the study, 386 female students (63.3%) and 224 male students (36.7%). The mean age was 20.58 ± 3.27 years. The rest of the demographic characteristics are presented in Table 1.

**Table 1:** Demographic characteristics

**Age**(mean, SD)	20.58 ± 3.27
**Gender** (n, %)	
Female	386 (63.3%)
Male	224 (36.7%)
**Semester** (n, %)	
1	16 (2.6%)
2	227 (37.2%)
3	49 (8.0%)
4	121 (17.4%)
5	20 (3.3%)
6	48 (7.9%)
7	39 (6.4%)
8	60 (9.8%)
9	30 (4.9%)
10	8 (1.3%)
11	1 (0.2%)
12	1 (0.2%)
**Field of study** (n, %)	
Arts and design	13 (2.1%)
Biological Sciences	22 (3.6%)
Humanitarian and political sciences	69 (11.3%)
Engineers	50 (8.2%)
Business Economics, administration, and accountancy	11 (1.8%)
Health sciences	445 (73%)

From the total sample, 436 students (71.5%) had not taken an online course before, and 174 students (28.5%) had taken an online course previously. When asked about their class preferences, 581 students (95.2%) preferred classroom classes, and 29 students (4.8%) preferred online classes.

When asked whether their perception of their academic performance was different after transitioning to online learning, 493 students (80.8%) perceived that their performance had worsened, 84 students (13.8%) reported that their performance had stayed the same as before, and 33 students (5.4%) reported that their performance had improved. When asked whether their grades were different since the transition to online learning, 296 students (48.5%) responded that their grades had stayed the same as before, 247 students (40.5%) reported worse grades compared with previous grades in classroom classes, and 67 students (11%) reported better grades than before.

The average ASCS score was 2.76 ± 0.35. The ASCS subscales were used to assess the Confidence and Effort categories. The total sample mean scores were 2.71 ± 0.37 for Confidence and 2.82 ± 0.45 for Effort. The total mean PHQ-9 score was 11.94 ± 6.90, and the mean GAD-7 score was 10.30 ± 5.66. For depression, 375 students (61.5%) scores higher than the cutoff score for possible depression diagnosis. As for anxiety, 318 students (52.1%) scores higher than the cutoff score for possible anxiety diagnosis. The depression and anxiety groups frequencies are presented in Table 2.

**Table 2:** ACSC Scores comparison according to depression and anxiety groups

Depression Groups	n (%)	Confidence Mean Scores	Effort Mean Scores	ASCS overall Scores
Minimal or none	103 (16.9%)	2.82 ± 0.41	2.87 ± 0.50	2.85 ± 0.42
Mild Depression Symptoms	132 (21.6%)	2.81 ± 0.38	2.87 ± 0.43	2.84 ± 0.34
Moderate Depression Symptoms	168 (27.5%)	2.69 ± 0.32	2.82 ± 0.46	2.75 ± 0.35
Moderately Severe Depression Symptoms	110 (18.0%)	2.62 ± 0.34	2.79 ± 0.40	2.71 ± 0.30
Severe Depression Symptoms	97 (15.9%)	2.57 ± 0.37	2.73 ± 0.43	2.65 ± 0.32
**Anxiety Groups**				
Minimal or none	130 (21.3%)	2.81 ± 0.40	2.86 ± 0.46	2.84 ± 0.39
Mild Anxiety	210 (34.4%)	2.71 ± 0.35	2.80 ± 0.45	2.75 ± 0.35
Moderate Anxiety	146 (23.9%)	2.66 ± 0.32	2.81 ± 0.42	2.73 ± 0.31
Severe Anxiety	124 (20.3%)	2.64 ± 0.41	2.82 ± 0.45	2.73 ± 0.36
**Co-diagnosis groups**				
Both depression and anxiety symptoms	447 (73.3%)	2.67 ± 0.36	2.80 ± 0.44	2.74 ± 0.34
Depression symptoms only	60 (9.8%)	2.80 ± 0.36	2.83 ± 0.38	2.81 ± 0.31
Anxiety only	33 (5.4%)	2.79 ± 0.38	2.85 ± 0.45	2.82 ± 0.36
Neither anxiety nor depression symptoms	70 (11.5%)	2.83 ± 0.43	2.89 ± 0.53	2.86 ± 0.44

Data is presented as mean scores and standard deviation

Student's T-test for independent samples was employed to contrast PHQ-9 and GAD-7 scores with the student’s perception of their performance. The groups' mean and *t* scores are presented in Table 3.

**Table 3:** Perceived academic performance and grade differneces groups comparisons in PHQ-9 and GAD-7 mean scores

Perceived academic performance differences	PHQ-9 mean scores	*t* scores	GAD-7 mean scores	*t* scores
Better performance	11.30 ± 8.31	-0.86	10.72 ± 6.35	0.46
Worse Performance	12.58 ± 6.70		10.68 ± 5.54	
**Grade differences**				
Better grades	11.65 ± 6.69	-2.54**	9.95 ± 5.53	-2.55**
Worse grades	14.04 ± 6.77		11.88 ± 5.47	

Data is presented as mean scores and standard deviation; t scores were obtained with Student's t-test. ** = *p* < 0.010

**Fig. 1:**
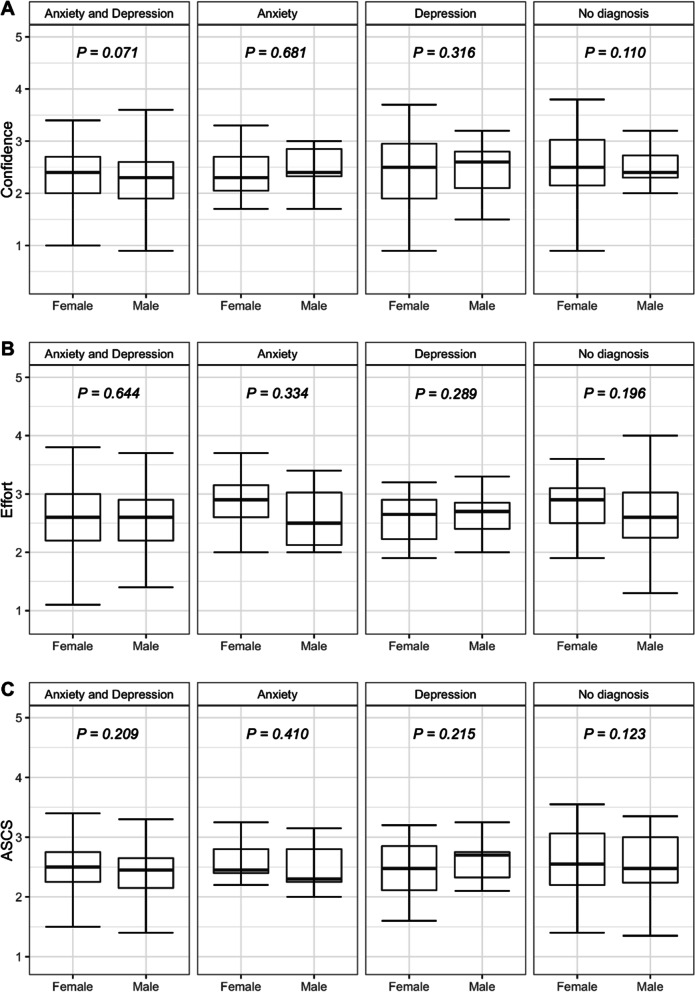
Gender differences in Confidence, Effort and ASCS mean scores in each diagnosis category

When genders were compared, female students had a higher PHQ-9 score and a greater risk of depression than male students; this difference was statistically significant (*p* = 0.039). Female students had higher GAD-7 scores than male students (*p* = 0.019), however when assessing risk for anxiety, both female and male students presented a similar risk. Female students scored higher on the ASCS mean, confidence, and effort scales. Gender comparisons can be found in Table 4. ACSC’s gender differences can be found in Fig. 1.

**Table 4:** Gender comparisons between mental health scores and academic self-concept

	**Female Students (*n* = 382)**	**Male Students (*n* = 224)**	***p value***
**PHQ-9 Mean Score**	12.29 ± 6.74	11.35 ± 7.15	0.104
**Depression Risk**			
* Yes*	248 (64.2%)	127 (56.7%)	0.039
* No*	138 (35.8%)	97 (43.3%)	
**GAD-7 Mean Score**	10.70 ± 5.59	9.60 ± 5.55	0.019
**Anxiety Risk**			
* Yes*	203 (52.3%)	115 (51.3%)	0.415
* No*	183 (47.4%)	109 (48.7%)	
**ASCS Mean Score**	2.78 ± 0.35	2.72 ± 0.35	0.048
* Confidence Mean Score*	2.73 ± 0.37	2.66 ± 0.37	0.030
* Effort Mean Score*	2.84 ± 0.45	2.79 ± 0.44	0.188

One-way between-group ANOVA was used to compare the ASCS scores according to the severity of depression symptoms. There was a significant effect of the severity of depression symptoms on the ASCS scores (F (4,605) = 6.506, *p* < 0.001). Post hoc comparisons using the Tukey honestly significant difference (HSD) test indicated that ASCS mean score differed significantly between the mild depression symptoms group and the moderately severe and severe depression symptoms groups. The mean ASCS scores also differed significantly between the severe and moderately severe depression symptoms groups compared with the minimal to no depression group. These differences continued after adjusting for multiple comparisons using the Bonferroni Test. For the mean ASCS scores, the severity of anxiety differed between groups (F (3,606) = 2.631, *p* < 0.05). The ASCS score differed between the no anxiety group and the moderate and severe anxiety groups. Different results persisted even after adjusting for multiple comparisons using the Bonferroni test.

One-way between-group ANOVA was used to compare the mean ASCS Confidence and Effort subscale scores according to the severity of depression symptoms. There was a significant effect of the severity of depression symptoms on the Confidence score (F (4,609) = 10.290, *p* < 0.001). The mean Confidence scores differed significantly between the severe depression symptoms group and the minimal or no depression and mild depression symptoms groups. These differences also persisted after adjusting for multiple comparisons using the Bonferroni test. The mean Effort subscale scores did not differ significantly according to the severity of depression symptoms (F (4,609) = 1.924, *p* > 0.05).

One-way between-group ANOVA was used to compare the Confidence and Effort scores according to the severity of anxiety. There was a significant effect of anxiety on Confidence scores (F (3,609) = 5.808, *p* < 0.001). Post hoc comparisons using the Tukey HDS test indicated that the Confidence score differed significantly between the no to minimal anxiety group and the moderate and severe anxiety groups. These differences continued after adjusting for multiple comparisons using the Bonferroni test. The mean Effort subscale scores did not differ significantly according to the severity of anxiety (F (3,609) = 0.606, *p* > 0.05).

## Discussion

Currently, the COVID-19 pandemic is a public health emergency of international concern, and governments have had to improvise strategies to keep afloat essential sectors such as education. Because of the conditions caused by the pandemic, UNESCO supports countries in their efforts to reduce the immediate impact of school closures and facilitate the continuity of education for all through distance learning, such as online learning programs [19]. In the USA, student preference for fully online courses has increased in recent years [36], but the student population in Mexico does not seem comfortable with the idea of distance education, and most students still prefer face-to-face academic programs [37]. Rural and indigenous communities have been disproportionately impacted by disparities in infrastructure and economic resources, with less than half of the population having access to the internet [37]. The Mexican authorities proposed an alternative of broadcasting elementary and high school classes via open television or radio. This did not, however, completely close the education gap created by the pandemic [38]. Additionally, as many students may be struggling psychologically because of the overall situation, it could be challenging for most students to maintain the same enthusiasm for online classes as for the face-to-face classroom environment.

Despite being a handy tool, it is essential to remember that the online modality of education also has some setbacks. For starters, not all students or university faculty are technologically skillful to access or manage the conference tools or online classrooms platforms available, complicating the process for both the lecturer and the students [39]. Additionally, for this modality to work, they all must have access to a computer or a laptop and a fast or stable internet connection as most classes are live. Orellana et al. defined one of the most critical challenges of virtual education for both students and teachers as the lack of interest and enthusiasm. The authors mention that students must be self-motivated, as being outside a classroom could alter their attention span and be susceptible to distractions outside the class. Also, both faculty members and students must acquire quickly the technological skills needed to use the classes materials successfully [40].

Some possible content-related issues include the role of instructors in content development and the integration of multimedia. Instructors must modify their teaching style, time management, and adaptation to the transition from face-to-face to virtual teaching. The absence of programs to prepare for this educational transition can directly impact the education of students, and in some cases, increase the level of burnout experienced by teachers [41]. Therefore, students must understand and take full responsibility for their learning needs, goals, and strategies [26]. Contrary to our results, Olmes et al. found a high acceptance of online courses. Although presenting a small sample of medical students, almost 3/4 of their sample were looking forward to receiving more online courses. Most students referred to miss actual patients during classes and commented that online classes could not replace patient contact in the hospital preferred traditional lectures [42]. Our sample not only preferred presential classes, but they also perceived a lower performance and grades when compared to the pre-pandemic era.

COVID-19's sudden outbreak inevitably resulted in widespread anxiety, depression, and other stress reactions in the general population, as a result of restrictions on daily life and social activities for an unknown period [9, 43]. Our study found that more than 80% of our student sample were had risk for mild to severe anxiety and depression after the beginning of the COVID-19 outbreak. Similar to Escalera-Chávez et al. [44], where at least 85% of their student sample suffered from anxiety and depression. This represents a significant increase over the baseline of 64% reported a year prior to the pandemic [45]. Our samples' risk of depression and anxiety is greater than that reported by a study conducted in the United States one month after the state of emergency was declared, in which at least one-third of young adults reported clinically elevated levels of depression and anxiety [46]. A study of 7,143 students in China reported that 24.9% experienced some degree of anxiety: 0.9% experienced severe anxiety, 2.7% moderate anxiety, and 21.3% mild anxiety [47]. Our sample had a higher prevalence of anxiety; most students reported experiencing anxiety, and more than one-fifth of the students met the cutoff scores for each category of mild, moderate, and severe anxiety. Our findings are consistent with that of a study of Spaniard university students in which 34.1% of participants reported moderate to severe depression, 21.3% reported moderate to severe anxiety symptoms, and 50.4% of their symptoms were related to a moderate or severe psychological impact during the outbreak and lockdown [48]. This finding may be explained by Mexico's cultural heritage, which is influenced by a collectivist mindset. Our culture is predisposed to seek refuge in a social group, whether that group is a family, a group of friends, or a classroom. This is in stark contrast to the populations of the United States, Central Europe, and Asia, which exhibit a more individualistic mindset [49]. Only 11% of our students were found to be at no or low risk of developing mental health disorders, an alarmingly low rate that demonstrates the critical nature of providing psychological resources to address social isolation during the pandemic until normal activity resumes.

Our sample reported lower academic self-concept scores than those reported by first-year college students [50]. When exploring the students' mental state in our sample, we found that the higher the PHQ-9 or GAD-7 scores, the lower the ASCS total scores. However, we also found that the participants who met the cutoff scores for moderate anxiety and severe anxiety for the GAD-7 scores presented similar scores in the Effort and Confidence subscales and ASCS overall scores. Although the students with moderate and severe anxiety had lower scores than those with no or minimal anxiety, it seems that the higher levels of anxiety are associated with a similar level of effort. Some students noted that anxiety and stress often inspired them to put in greater effort in their studies. In a study of nursing students that assessed the impact of stress factors and depressive symptoms on academic performance, higher stress rates were associated with better individual semester performance, which suggests a positive impact of stress on academic performance [51]. Nevertheless, academic performance may be more susceptible because depressive symptoms can interfere with intellectual activity, contributing to school failure [52]. Our findings are consistent with previous research, as most of our sample reported that their academic performance deteriorated at the start of the online classes, they perceived receiving lower grades, and a higher risk of depressive symptoms was associated with lower academic performance [51, 52]. While some authors reported high levels of acceptance for distance learning [22, 24, 53], some students from fields such as veterinary medicine, nursing or medicine reported high levels of acceptance for the program's theoretical components, but low ratings for the program's practical components [23, 54, 55].

### Limitations

Our study has a number of methodological shortcomings. To begin, the scales used in this study were not developed for the purpose of screening for depression or anxiety associated with COVID-19. Because this study was conducted at the start of the pandemic, it was necessary to conduct it using generalized mental health screening questionnaires. Additionally, this study began during the implementation of a strict social isolation policy, which may produce results that differ from current research using more flexible social norms. Another limitation of our study is that it is based on a single location's sample of university students, which may make generalizing the findings difficult. Fortunately, the study's universities enroll students from across the country, which may help mitigate this bias.

## Conclusion

The COVID-19 pandemic has directly impacted students' performance and the ability to receive information. Like most countries, Mexico has had to improvise strategies to continue providing education at all levels. These strategies have addressed the operational aspects, but the psychological impact of confinement and other factors related to the pandemic has led to an increase in students' incidence of depression and anxiety. This trend may contribute to barriers that prevent proper learning and affect students' preparation, grades, and performance. Strategies should be created to support students continue their academic program through distance learning as social isolation is used to limit the possible spread of disease. However, this must be delivered in a way that avoids imposing barriers to learning. Such strategies must incorporate an understanding of how stressful and complicated social isolation can be.

## Data Availability

The datasets used and/or analyzed during the current study are available from the corresponding author on reasonable request.

## Abbreviations

ANOVA: Analysis Of Variance
ASCS: Academic Self-Concept Scale
COVID-19: Coronavirus Disease-19
GAD-7: General Anxiety Disorder-7
HSD: Honestly Significant Difference
PHQ-9: Patient Health Questionnarie-9
PTSD: Post-Traumatic Stress Disorder
SARS: Severe Acute Respiratory Syndrome
UNESCO: Educational, Scientific, and Cultural organization

## References

[1] Rajkumar RP. COVID-19 and mental health: A review of the existing literature. Asian J Psychiatr. 2020;52:102066. 10.1016/j.ajp.2020.102066. 10.1016/j.ajp.2020.102066 PMC7151415 32302935

[2] Pfefferbaum B, Carol SN. Mental Health and the Covid-19 Pandemic. N Engl J Med. 2020;383(6):510-512. 10.1056/NEJMp2008017 32283003

[3] McEwen BS, Morrison JH (2013). The Brain on Stress: Vulnerability and Plasticity of the Prefrontal Cortex over the Life Course. Neuron.

[4] Lam MHB, Wing YK, Yu MWM, Leung CM, Ma RCW, Kong APS (2009). Mental morbidities and chronic fatigue in severe acute respiratory syndrome survivors long-term follow-up. Arch Intern Med.

[5] Garcia-Priego BA, Triana-Romero A, Pinto-Galvez SM, Duran-Ramos C, Salas-Nolasco O, Reyes MM, et al. Anxiety, depression, attitudes, and internet addiction during the initial phase of the 2019 coronavirus disease (COVID-19) epidemic: A cross-sectional study in Mexico. medRxiv. 2020;2020.05.10.20095844.

[6] García-Reyna B, Castillo-García GD, Barbosa-Camacho FJ, Cervantes-Cardona GA, Cervantes-Pérez E, Torres-Mendoza BM, et al. Fear of COVID-19 Scale for Hospital Staff in Regional Hospitals in Mexico: a Brief Report. Int J Ment Health Addict. 2022;20(3):1687–94. 10.1007/s11469-020-00413-x PMC7640993 33169075

[7] Barbosa-Camacho FJ, García-Reyna B, Cervantes-Cardona GA, Cervantes-Pérez E, Chavarria-Avila E, Pintor-Belmontes KJ, et al. Comparison of Fear of COVID-19 in Medical and Nonmedical Personnel in a Public Hospital in Mexico. Int J Ment Health Addict. 2021;1–12. 10.1007/s11469-021-00600-4. 10.1007/s11469-021-00600-4 PMC8324181 34366729

[8] Ahorsu DK, Lin C, Imani V, Saffari M, Griffiths MD, Pakpour AH. The Fear of COVID-19 Scale : Development and Initial Validation. Int J Mental Health Addict. 2022;20(3):1537–45. 10.1007/s11469-020-00270-8 PMC7100496 32226353

[9] Wang C, Pan R, Wan X, Tan Y, Xu L, McIntyre RS, et al. A longitudinal study on the mental health of general population during the COVID-19 epidemic in China. Brain Behav Immun. 2020;87:40–48. 10.1016/j.bbi.2020.04.028. 10.1016/j.bbi.2020.04.028 PMC7153528 32298802

[10] Lee SA. Coronavirus anxiety scale: A brief mental health screener for COVID-19 related anxiety. Death Stud. 2020;44(7):393–401. 10.1080/07481187.2020.1748481 32299304

[11] Lee SA, Mathis AA, Jobe MC, Pappalardo EA. Clinically significant fear and anxiety of COVID-19: A psychometric examination of the Coronavirus Anxiety Scale. Psychiatry Res. 2020;290:113112. 10.1016/j.psychres.2020.113112. 10.1016/j.psychres.2020.113112 PMC7237368 32460185

[12] Gobierno de Jalisco. Criterios para la activación del botón de emergencia [Criteria for the emergency button activation]. 2020. https://coronavirus.jalisco.gob.mx/criterios-para-la-activacion-del-boton-de-emergencia/.

[13] Prensa. Con el objetivo de salvar vidas, reducir los contagios y evitar el colapso de hospitales, las medidas de aislamiento social y el uso de cubrebocas en la calle serán obligatorios en Jalisco [In order to save lives, reduce contagion and avoid the collapse. Gobierno del Estado de Jalisco. 2020. https://www.jalisco.gob.mx/es/prensa/noticias/103420.

[14] Zeng Q, Liang Z, Zhang M, Xia Y, Li J, Kang D, et al. Impact of Academic Support on Anxiety and Depression of Chinese Graduate Students During the COVID-19 Pandemic: Mediating Role of Academic Performance. Psychol Res Behav Manag. 2021;14:2209–19. 10.2147/PRBM.S345021. 10.2147/PRBM.S345021 PMC8722691 35002339

[15] Son C, Hegde S, Smith A, Wang X, Sasangohar F. Effects of COVID-19 on College Students’ Mental Health in the United States: Interview Survey Study. J Med Internet Res . 2020;22(9):e21279. 10.2196/21279. 10.2196/21279 PMC7473764 32805704

[16] Quintero López C, Vera-Gil DV (2021). Depression in university students derived from Covid-19: a classification model. Cuadernos Hispanoamericanos de Psicología.

[17] Awadalla S, Davies EB, Glazebrook C. A longitudinal cohort study to explore the relationship between depression, anxiety and academic performance among Emirati university students. 2020;20(1):448. 10.1186/s12888-020-02854-z. 10.1186/s12888-020-02854-z PMC7488388 32917172

[18] Pragholapati A. COVID-19 Impact on Students. EdArXiv Preprints. 2020. 10.35542/osf.io/895ed.

[19] UNESCO. School closures caused by Coronavirus (Covid-19). UNESCO. 2020. https://en.unesco.org/covid19/educationresponse.

[20] Redacción. Publica DOF acuerdo de suspensión de clases a nivel nacional por Covid-19 [DOF publishes agreement to suspend classes at the national level due to Covid-19]. La Jornada. 2020. https://www.jornada.com.mx/ultimas/sociedad/2020/03/16/publica-dof-acuerdo-de-suspension-de-clases-a-nivel-nacional-por-covid-19-5707.html.

[21] Chick RC, Clifton GT, Peace KM, Propper BW, Hale DF, Alseidi AA, et al. Using Technology to Maintain the Education of Residents During the COVID-19 Pandemic. J Surg Educ. 2020;77(4):729–32. 10.1016/j.jsurg.2020.03.018 PMC7270491 32253133

[22] Vargas-Ramos JC, Lerma C, Guzmán-Saldaña RME, Lerma A, Bosques-Brugada LE, González-Fragoso CM. Academic Performance during the COVID-19 Pandemic and Its Relationship with Demographic Factors and Alcohol Consumption in College Students. Int J Environ Res Public Health. 2021;19(1):365. 10.3390/ijerph19010365. 10.3390/ijerph19010365 PMC8744874 35010625

[23] Oducado RMF, Estoque H. Online Learning in Nursing Education During the COVID-19 Pandemic: Stress, Satisfaction, and Academic Performance. J Nurs Pract. 2021;4:143–53. 10.30994/jnp.v4i2.128.

[24] Iglesias-Pradas S, Hernández-García Á, Chaparro-Peláez J, Prieto JL. Emergency remote teaching and students’ academic performance in higher education during the COVID-19 pandemic: A case study. Comput Human Behav. 2021;119:106713. 10.1016/j.chb.2021.106713. 10.1016/j.chb.2021.106713 PMC8631572 34866769

[25] Brockfeld T, Müller B, Laffolie J de. Video versus live lecture courses: a comparative evaluation of lecture types and results. Med Educ Online. 2018;23(1):1555434. 10.1080/10872981.2018.1555434. 10.1080/10872981.2018.1555434 PMC6300084 30560721

[26] Kebritchi M, Lipschuetz A, Santiague L. Issues and Challenges for Teaching Successful Online Courses in Higher Education: A Literature Review. J Educ Technol Syst. 2017;46:4–29. 10.1177/0047239516661713.

[27] Liu WC, Wang CKJ. Academic self-concept: A cross-sectional study of grade and gender differences in a Singapore secondary school. Asia Pac Educ Rev. 2005;6(1):20–7.

[28] Baxter AJ, Scott KM, Vos T, Whiteford HA. Global prevalence of anxiety disorders: A systematic review and meta-regression. Psychol Med. 2013;43(5):897–910. 10.1017/S003329171200147X 22781489

[29] Ojeda-Torres D, González-González C, Cambero-González E, Madrigal-De-León E, González-Méndez J, Calderón-Rivera D. Prevalencia de los Trastornos Mentales y la infraestructura en Salud Mental en el Estado de Jalisco. Salud Jalisco. 2019;6 Esp:6–15.

[30] Liu WC, Wang CKJ. The effects of perceived home environment and classroom climate on male and female students academic self-concept. J Educ. 2007;5(1):52–71.

[31] Granero-Gallegos A, Baena-Extremera A, Escaravajal JC, Baños R. Validation of the Academic Self-Concept Scale in the Spanish University Context. Educ Sci. 2021;11(10):653. 10.3390/educsci11100653.

[32] Kroenke K, Spitzer RL, Williams JBW. The PHQ-9: Validity of a brief depression severity measure. J Gen Intern Med . 2001;16(9):606-13. 10.1046/j.1525-1497.2001.016009606.x PMC1495268 11556941

[33] Gilbody S, Richards D, Brealey S, Hewitt C. Screening for depression in medical settings with the Patient Health Questionnaire (PHQ): a diagnostic meta-analysis. J Gen Intern Med. 2007;22(11):1596-602. 10.1007/s11606-007-0333-y PMC2219806 17874169

[34] Löwe B, Decker O, Müller S, Brähler E, Schellberg D, Herzog W, et al. Validation and standardization of the Generalized Anxiety Disorder Screener (GAD-7) in the general population. Med Care. 2008;46(3):266-74. 10.1097/MLR.0b013e318160d093 18388841

[35] Spitzer RL, Kroenke K, Williams JBW, Löwe B. A Brief Measure for Assessing Generalized Anxiety Disorder: The GAD-7. Arch Intern Med. 2006;166(10):1092–7. 10.1001/archinte.166.10.1092 16717171

[36] Ortagus JC (2017). From the periphery to prominence: An examination of the changing profile of online students in American higher education. Internet Higher Educ.

[37] Monroy-Gómez-Franco L, Vélez-Grajales R, López-Calva LF. The potential effects of the COVID-19 pandemic on learnings. Int J Educ Dev. 2022;91:102581. 10.1016/j.ijedudev.2022.102581. 10.1016/j.ijedudev.2022.102581 PMC8920787 35308115

[38] Rivers M, Suarez K, Gallón N. Mexico launches school broadcasts on television and radio for kids. CNN. 2020. https://edition.cnn.com/2020/08/22/americas/mexico-covid-19-classes-on-tv-intl/index.html. Accessed 18 Apr 2022.

[39] Sahu P, Closure of Universities Due to Coronavirus Disease,. (COVID-19): Impact on Education and Mental Health of Students and Academic Staff. Cureus. 2020;12(4):e7541. 10.7759/cureus.7541. 10.7759/cureus.7541 PMC7198094 32377489

[40] Orellana-Parapi JM, Maesaroh LI, Basuki B, Masykuri ES. Virtual Education: A Brief Overview of Its Role in the Current Educational System. Scripta Eng Depart J. 2020;7:8–11. 10.37729/scripta.v7i1.632.

[41] Gutentag T, Orner A, Asterhan CSC. Classroom discussion practices in online remote secondary school settings during COVID-19. Comput Human Behav. 2022;132:107250. 10.1016/j.chb.2022.107250. 10.1016/j.chb.2022.107250 PMC8881193 35250162

[42] Olmes GL, Zimmermann JSM, Stotz L, Takacs FZ, Hamza A, Radosa MP, et al. Students’ attitudes toward digital learning during the COVID-19 pandemic: a survey conducted following an online course in gynecology and obstetrics. Arch Gynecol Obstet. 2021;304(4):957-963. 10.1007/s00404-021-06131-6. 10.1007/s00404-021-06131-6 PMC8341044 34355284

[43] Henssler J, Stock F, van Bohemen J, Walter H, Heinz A, Brandt L. Mental health effects of infection containment strategies: quarantine and isolation—a systematic review and meta-analysis. Eur Arch Psychiatry Clin Neurosc . 2021;271(2):223-34. 10.1007/s00406-020-01196-x PMC7538183 33025099

[44] Escalera-Chávez ME, Santana JC, García-Santillán A. Do Coronavirus Confinement Measures Cause Anxiety, Stress and Depression in University Students?. Eur J Educ Res. 2021;10:855–64. 10.12973/eu-jer.10.2.855.

[45] Almonte-Becerril M, Parra-Torres N-M, Baltazar-Pedro MF. Prevalence of signs of depression and its relationship with academic performance in students of the Intercultural University of the State of Puebla, Mexico. [Prevalencia de signos de depresión y su relación con el desempeño académico en Alumnos de la Universidad Intercultural del Estado de Puebla, México]. Holopraxis. 2019;3:140-55.

[46] Liu CH, Zhang E, Wong GTF, Hyun S, Hahm H “Chris.” Factors associated with depression, anxiety, and PTSD symptomatology during the COVID-19 pandemic: Clinical implications for U.S. young adult mental health. Psychiatry Res. 2020;290:113172. 10.1016/j.psychres.2020.113172. 10.1016/j.psychres.2020.113172 PMC7263263 32512357

[47] Cao W, Fang Z, Hou G, Han M, Xu X, Dong J, et al. The psychological impact of the COVID-19 epidemic on college students in China. Psychiatry Res. 2020;287:112934. 10.1016/j.psychres.2020.112934. 10.1016/j.psychres.2020.112934 PMC7102633 32229390

[48] Odriozola-González P, Planchuelo-Gómez Á, Irurtia MJ, de Luis-García R. Psychological effects of the COVID-19 outbreak and lockdown among students and workers of a Spanish university. Psychiatry Res. 2020;290:113108. 10.1016/j.psychres.2020.113108. 10.1016/j.psychres.2020.113108 PMC7236679 32450409

[49] Gomez C, Taylor KA. Cultural differences in conflict resolution strategies: A US–Mexico comparison. 2017;18:33–51. 10.1177/1470595817747638.

[50] Haktanir A, Watson JC, Ermis-Demirtas H, Karaman MA, Freeman PD, Kumaran A, et al. Resilience, Academic Self-Concept, and College Adjustment Among First-Year Students. J Coll Stud Retent: Res Theory Pract. 2021;23(1):161-178. 10.1177/1521025118810666.

[51] Ferreira da Fonseca JR, Siqueira Costa Calache AL, Rodrigues dos Santos M, Marques da Silva R, Alvarez Moretto S. Association of stress factors and depressive symptoms with the academic performance of nursing students. Rev Esc Enferm USP. 2019;53:03530. 10.1590/S1980-220X2018030403530. 10.1590/S1980-220X2018030403530 31800821

[52] Rojas García A, Ruggero C. Depresión, ansiedad y rendimiento académico en estudiantes universitarios. Revista Intercontinental de Psicología y Educación. 2013;15(1):47–60.

[53] Zhang W, Gu J, Li F, Feng F, Chen H, Xing X, et al. The effect of flipped classroom in multiple clinical skills training for clinical interns on Objective Structured Clinical Examinations (OSCE). 2022;27(1):2013405. 10.1080/10872981.2021.2013405. 10.1080/10872981.2021.2013405 PMC8676640 34898400

[54] Mahdy MAA. The Impact of COVID-19 Pandemic on the Academic Performance of Veterinary Medical Students. Frontiers in Veterinary Science. 2020;7:732. 10.3389/fvets.2020.594261. 10.3389/fvets.2020.594261 PMC7572855 33134368

[55] Mortagy M, Abdelhameed A, Sexton P, Olken M, Hegazy MT, Gawad MA, et al. Online medical education in Egypt during the COVID-19 pandemic: a nationwide assessment of medical students’ usage and perceptions. BMC Med Educ . 2022;22(1):218. 10.1186/s12909-022-03249-2. 10.1186/s12909-022-03249-2 PMC8966850 35354406

